# Development of central nervous system metastases as a first site of metastatic disease in breast cancer patients treated in the neoadjuvant trials GeparQuinto and GeparSixto

**DOI:** 10.1186/s13058-019-1144-x

**Published:** 2019-05-10

**Authors:** Elena Laakmann, Isabell Witzel, Peter A. Fasching, Mahdi Rezai, Christian Schem, Christine Solbach, Hans Tesch, Peter Klare, Andreas Schneeweiss, Christoph Salat, Dirk-Michael Zahm, Jens-Uwe Blohmer, Barbara Ingold-Heppner, Jens Huober, Claus Hanusch, Christian Jackisch, Mattea Reinisch, Michael Untch, Gunter von Minckwitz, Valentina Nekljudova, Volkmar Müller, Sibylle Loibl

**Affiliations:** 1 Department of Gynecology, University Medical Center Hamburg-Eppendorf, Martinistrasse 52, 20246 Hamburg, Germany; 2 Department of Gynecology and Obstetrics, University Hospital Erlangen, Comprehensive Cancer Center Erlangen-EMN, Friedrich-Alexander University Erlangen-Nuremberg, Erlangen, Germany; 3 European Breast Center Duesseldorf, Luise-Rainer-Str. 6-10, 40235 Duesseldorf, Germany; 4 Department of Gynecology, University Medical Center Schleswig-Holstein, Campus Kiel, Arnold-Heller-Str. 3, Haus 24, 24105 Kiel, Germany; 5 Breastcancer Center Hamburg, Moorkamp 2–6, 20357 Hamburg, Germany; 6 Department of Gynecology, University Hospital Frankfurt, Theodor-Stern-Kai 7, 60590 Frankfurt, Germany; 7 Center for Hematology und Oncology Bethanien Frankfurt, Im Prüfling 17-19, 60389 Frankfurt/Main, Germany; 8 Medical Center, Lichtenberg, Möllendorffstraße 52, 10367 Berlin, Germany; 9 National Center for Tumor Diseases, Division Gynecologic Oncology, University Hospital, Im Neuenheimer Feld 460, 69120 Heidelberg, Germany; 10 Medical Center for Hematology and Oncology, Winthirstr. 7, 80639 Munich, Germany; 11 Department of Gynecology, SRH Wald-Klinikum Gera gGmbH, Strasse des Friedens 122, 07548 Gera, Germany; 12 Department of Gynecology and Breast Cancer, Charité, Charitéplatz 1, 10117 Berlin, Germany; 13 Department of Pathology, Charité, Charitéplatz 1, 10117 Berlin, Germany; 14 Department of Gynecology, University of Ulm, Prittwitzstrasse 43, 89075 Ulm, Germany; 15 Department of Gynecology, Rotkreuzklinikum München, Taxisstraße 3, 80637 Munich, Germany; 16 Department of Obstetrics and Gynecology, Sana Klinikum Offenbach, Starkenburgring 66, 63069 Offenbach, Germany; 17 Breast Unit, Kliniken Essen-Mitte Evang. Huyssens-Stiftung/Knappschaft GmbH, Henricistrasse 92, 45136 Essen, Germany; 18 Department of Gynecology, HELIOS Klinikum Berlin-Buch, Schwanebecker Chaussee 50, 13125 Berlin, Germany; 19 German Breast Group GmbH, Martin Behaim Strasse 12, 63263 Neu-Isenburg, Germany

**Keywords:** Central nervous system metastases, Breast cancer, First site of metastatic disease

## Abstract

**Background:**

The incidence of central nervous system (CNS) metastases in breast cancer patients is rising and has become a major clinical challenge. Only few data are published concerning risk factors for the development of CNS metastases as a first site of metastatic disease in breast cancer patients. Moreover, the incidence of CNS metastases after modern neoadjuvant treatment is not clear.

**Methods:**

We analyzed clinical factors associated with the occurrence of CNS metastases as the first site of metastatic disease in breast cancer patients after neoadjuvant treatment in the trials GeparQuinto and GeparSixto (*n* = 3160) where patients received targeted treatment in addition to taxane and anthracycline-based chemotherapy.

**Results:**

After a median follow-up of 61 months, 108 (3%) of a total of 3160 patients developed CNS metastases as the first site of recurrence and 411 (13%) patients had metastatic disease outside the CNS. Thirty-six patients (1%) developed both CNS metastases and other distant metastases as the first site of metastatic disease. Regarding subtypes of the primary tumor, 1% of luminal A-like (11/954), 2% of luminal B-like (7/381), 4% of HER2-positive (34/809), and 6% of triple-negative patients (56/1008) developed CNS metastases as the first site of metastatic disease.

In multivariate analysis, risk factors for the development of CNS metastases were larger tumor size (cT3–4; HR 1.63, 95% CI 1.08–2.46, *p* = 0.021), node-positive disease (HR 2.57, 95% CI 1.64–4.04, *p* < 0.001), no pCR after neoadjuvant chemotherapy (HR 2.29, 95% CI 1.32–3.97, *p* = 0.003), and HER2-positive (HR 3.80, 95% CI 1.89–7.64, *p* < 0.001) or triple-negative subtype (HR 6.38, 95% CI 3.28–12.44, *p* < 0.001).

**Conclusions:**

Especially patients with HER2-positive and triple-negative tumors are at risk of developing CNS metastases despite effective systemic treatment. A better understanding of the underlying mechanisms is required in order to develop potential preventive strategies.

## Background

Central nervous system (CNS) metastases in breast cancer patients are a clinically relevant problem. CNS metastases are associated with shorter survival and impaired quality of life compared with extracranial metastases. Despite the use of neurosurgery and radiotherapy, only the minority of patients survives longer than 1 year [1].

Depending on tumor subtype, survival times of 3.7–15 months after the occurrence of CNS metastases have been described [2]. As in the primary tumor setting, patients with triple-negative breast cancer (TNBC) have the worst prognosis. In a retrospective study by Niikura et al. with 1256 patients diagnosed with brain metastases, the median overall survival of TNBC patients after the CNS metastasis diagnosis was 4.9 months and that of human epidermal growth factor receptor 2 (HER2)-positive patients was 11.5 months [3]

In the treatment of non-metastatic breast cancer, relevant improvement has been achieved in the last years resulting in prolonged survival. Neoadjuvant systemic treatment has become a standard procedure for the treatment of primary breast cancer. Pathologic complete response (pCR) rates after neoadjuvant chemotherapy are increasing with current treatment standards and are reflected by an improved patient outcome. pCR rates of 58% for HER2 positive and 37% for TNBC could be achieved [4]. A 5-year breast cancer-specific survival rate of 96% and 75% was observed in an analysis of Boughey et al. for HER2-positive and TNBC patients, respectively [5].

However, about 4% of HER2-positive and 7% of TNBC patients develop CNS metastases after the adjuvant treatment (5-year cumulative incidence of CNS metastases) in historic cohorts [6], and it remains unclear which patients are at high risk for the development of CNS metastases after neoadjuvant treatment with the current standard.

So far, limited insight is available into the biology of CNS metastases in breast cancer patients. It could be assumed that patients who develop CNS metastases as a first site of metastatic disease have distinct clinical or tumor features and that these might help to better understand factors that are a predisposition for CNS metastases. However, only limited studies are published concerning risk factors for the development of CNS metastases. Moreover, most studies on this topic evaluated cohorts with only a small number of patients that did not receive treatment regimens as of the current standard. This is of relevance since modern targeted therapy could potentially influence the pattern of metastatic spread. We therefore investigated the clinical factors associated with the occurrence of CNS metastases as the first site of metastatic disease in 3160 breast cancer patients after modern neoadjuvant systemic therapy.

The preliminary results of our evaluation were presented at the San Antonio Breast Cancer Symposium in 2017 [7].

## Methods

The clinical data for the analysis were derived from the neoadjuvant trials GeparQuinto and GeparSixto of the German Breast Group, consisting of 3160 patients, who have been treated with neoadjuvant therapy for early breast cancer.

Briefly, in GeparQuinto, patients with primary HER2-positive breast cancer (*n* = 615) received either lapatinib or trastuzumab in addition to an anthracycline and taxane-based therapy, patients with HER2-negative breast cancer (*n* = 1925) received an anthracycline and taxane-containing regimen and were randomly assigned to receive bevacizumab (*n* = 956), and those not responding after four cycles of anthracyclines (*n* = 395) received paclitaxel (*n* = 198) or paclitaxel plus everolimus (*n* = 197) [8–10].

In GeparSixto, patients with previously untreated, non-metastatic, TNBC (*n* = 315) and HER2-positive (*n* = 273) breast cancer were treated with paclitaxel and non-pegylated liposomal doxorubicin. Patients with triple-negative breast cancer received simultaneous bevacizumab. Patients with HER2-positive disease received simultaneous trastuzumab and lapatinib. Patients were randomly assigned to receive, at the same time as the backbone regimens, either carboplatin or no carboplatin [11].

Written informed consent was obtained from all patients before enrolment in the GeparQuinto and GeparSixto trials.

### Statistics

The primary objective of our analysis was to assess the influence of the baseline characteristics and pCR on the occurrence of CNS metastases as the first site of metastatic disease (with or without simultaneous occurrence of other metastases) in both trials mentioned above. It was done by assessing time (in months, from randomization in the study) to the occurrence of the CNS metastasis as the first site of metastatic disease according to the competing risk model of Fine and Gray [12], and cumulative incidence function was presented graphically; other distant metastases, contralateral breast cancer, secondary malignancies, or death before any event were considered competing events.

The following covariates were included into a Fine-Gray model: age in years (continuous), tumor stage (cT1–2 vs. cT3–4), nodal status (cN0 vs. cN+), primary tumor subtype (TNBC, HER2 positive/negative, luminal (hormone receptor-positive, HER2-negative) A-like (grades 1–2) and B-like (grade 3)), and study (to adjust for possible heterogeneity). Additionally, a multivariate Fine-Gray model including covariates above and pCR was performed to explore what portion of the effect is mediated by pCR; to avoid guarantee-time bias [13], a landmark of 24 weeks for GeparQuinto patients and 18 weeks for GeparSixto patients was used.

Secondary endpoints were as follows:

- To assess time (in months, from randomization in the study) to the occurrence of the non-CNS metastasis as the first site of relapse (and to compare to the time to occurrence of the CNS metastases as the first site of metastatic disease). CNS metastases, contralateral breast cancer, secondary malignancies, or death before any event were considered competing events. The cumulative incidence functions for CNS and non-CNS metastases were presented graphically.
- To describe the first metastatic site at the first distant relapse. The data concerning the first metastatic site was collected in the context of follow-up investigations.

The following categories were assessed:

- If a patient had CNS metastasis as a first event (yes/no), if CNS metastases were the only localization (yes/no), or if CNS metastases were diagnosed simultaneously with other metastases (skin, bone, liver, lung/pleura, non-locoregional lymph node, or other localization)
- If a patient had non-CNS metastases as a first event. The following categories of the metastasis localization could be documented: skin, bone, liver, lung/pleura, non-locoregional lymph node, or other localization

The statistical analyses were performed with SAS 9.2 under SAS Enterprise Guide 4.3 and with SAS 9.4 (for competing risk analyses).

## Results

### Patient’s characteristics

A total of 3160 patients treated in GeparQuinto and GeparSixto trials were available for the analysis. Two thousand five hundred seventy-two patients were treated within the GeparQuinto, and 588 within the GeparSixto trial. Seventy-three percent (*n* = 2306) of the patients had a cT1 tumor stage before neoadjuvant chemotherapy. Fifty-one percent (*n* = 1581) had a node-negative, and 49% (*n* = 1533) a node-positive (cN1-3) primary breast cancer. Concerning the tumor subtype, 32% (*n* = 1008) had a TNBC, 30% (*n* = 954) a luminal A-like, 12% (*n* = 381) a luminal B-like, and 26% (*n* = 809) HER2-positive tumor. pCR after neoadjuvant treatment was observed in 23% (*n* = 738) of the patients. The highest pCR rate was observed in patients with TNBC (38%, *n* = 379). Patients with HER2-positive tumors had a pCR rate of 30% (*n* = 245), and luminal A- and B-like breast cancer patients had pCR rates of 6% (*n* = 61) and 14% (*n* = 52), respectively (Table 1).

**Table 1:** pCR in the overall cohort according to breast cancer subtypes

Breast cancer subtype	Patients without pCR (*n*, %)	Patients with pCR (*n*,%)
Luminal A-like	893 (93.6)	61 (6.4)
Luminal B-like	329 (86.4)	52 (13.6)
HER2 positive	564 (69.7)	245 (30.3)
TNBC	629 (62.4)	379 (37.6)

### Development of metastases (CNS metastases and non-CNS metastases)

**Fig. 1:**
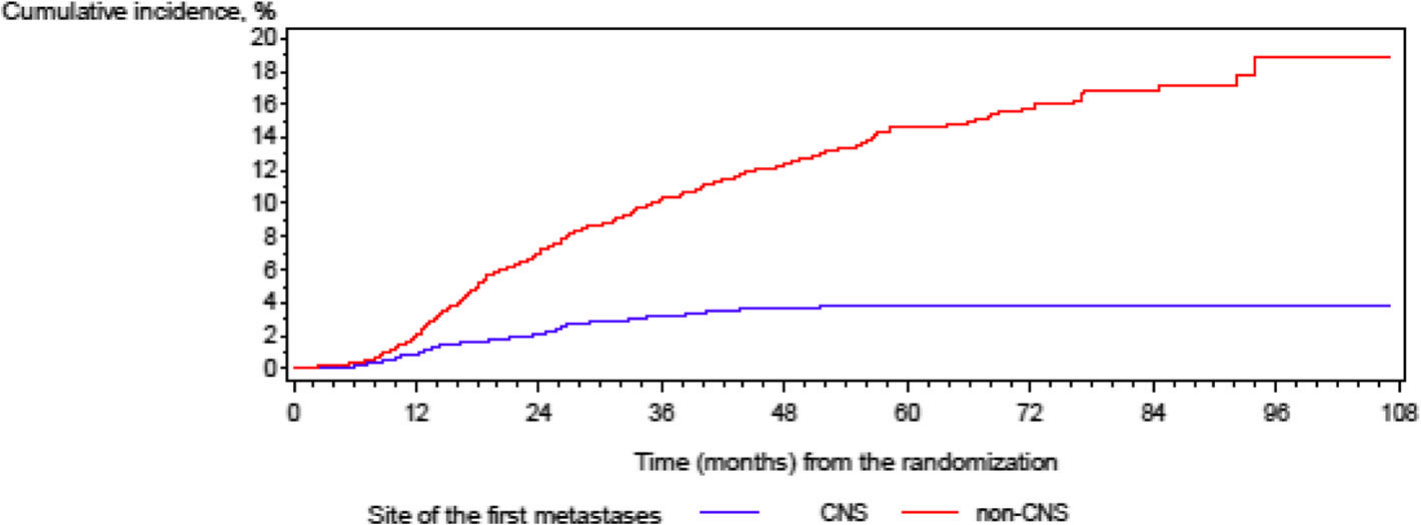
Cumulative incidence of metastases

After a median follow-up of 61 months (IQR 45–73), 108 (3%) of a total of 3160 patients developed CNS metastases as the first site of metastatic disease, 411 (13%) patients had distant metastases outside the brain as the first site of metastatic disease, and 2641 (84%) of the patients had no metastatic disease (Fig. 1: cumulative incidence of metastases). A total of 72 patients (2%) had CNS metastases as the only localization of the metastatic disease, and 36 patients (1%) had other distant metastases in addition to CNS metastases.

CNS metastases as the first site of metastatic disease occurred less frequently than other metastases (5-year cumulative incidence of CNS metastases was 3.8%, and cumulative incidence of the metastases outside the brain was 14.6% according to the competing risk model, Fig. 1: cumulative incidence of metastases).

The median age of patients with CNS metastases as the first site of metastatic disease was 49 years (range 27–75). Seventy-nine percent of the patients (*n* = 85) were initially treated within GeparQuinto, and 21% (*n* = 23) within the GeparSixto study.

Of the patients with CNS metastases as the first site of metastatic disease, 57% (*n* = 61) had a cT1-2 tumor at the time of diagnosis, 73% (*n* = 79) node-positive disease (cN1–cN3), and 62% high grading (*n* = 67, G3 tumor) of the primary tumor. After neoadjuvant therapy, most of the patients had no pCR (*n* = 92, 85%).

Regarding subtypes of the primary tumor, 32% of the patients had HER2-positive (*n* = 34) and 52% (*n* = 56) TNBC subtype.

Patients with non-CNS metastases as the first site of metastatic disease had a median age of 48 years (range 21–76). Eighty-six percent of the patients (*n* = 352) were initially treated within GeparQuinto, and 14% (*n* = 59) within the GeparSixto study.

Sixty-three percent (*n* = 259) of the patients with non-CNS metastases as the first site of metastatic disease had a cT1–2 tumor at the time of diagnosis, 60% of the patients had node-positive disease (cN1–cN3, *n* = 244), and 52% had a high-grade tumor (*n* = 213 with a G3 tumor). Ninety-two percent of the patients in this group had no pCR after the neoadjuvant chemotherapy (*n* = 376). Regarding subtypes of the primary tumor, 19% of the patients in this group had HER2-positive (*n* = 76) and 40% (*n* = 165) TNBC subtype.

Concerning the site of the first metastatic disease (non-CNS metastases), of the 411 patients, 173 (42%) developed bone metastases, 157 (38%) developed liver metastases, 141 (34%) had lung/pleural metastases, 70 (17%) had lymphatic metastases in non-locoregional lymph nodes, and 39 (9%) developed skin metastases. Forty of 411 patients (10%) had other sites of the first metastatic disease (non-CNS metastases).

For the detailed summary concerning patient’s characteristics, see Table 2.

**Table 2:** Patient’s characteristics

Parameter	CNS metastases as the first site of metastatic disease, *N* = 108	Non-CNS metastases as the first site of metastatic disease, *N* = 411	Patients without distant relapse, *N* = 2641	Overall, *N* = 3160
Age, median (years)	48.5	48.0	49.0	48.0
cT (*n*, %)				
cT1–2	61 (57.0)	259 (63.2)	1986 (75.5)	2306 (73.3)
cT3	15 (14.0)	77 (18.8)	366 (13.9)	458 (14.5)
cT4a–c	5 (4.7)	26 (6.3)	118 (4.5)	149 (4.7)
cT4d	26 (24.3)	48 (11.7)	161 (6.1)	235 (7.5)
Missing	1	1	10	12
cN (*n*, %)				
cN0	28 (26.2)	159 (39.5)	1394 (53.5)	1581 (50.8)
cN1	68 (63.6)	209 (51.9)	1100 (42.2)	1377 (44.2)
cN2	9 (8.4)	23 (5.7)	90 (3.5)	122 (3.9)
cN3	2 (1.9)	12 (3.0)	20 (0.8)	34 (1.1)
Missing	1	8	37	46
Breast cancer subtype				
Luminal A-like (grades 1–2)	11 (10.2)	107 (26.2)	836 (31.7)	954 (30.3)
Luminal B-like (grade 3)	7 (6.5)	61 (14.9)	313 (11.9)	381 (12.1)
HER2+	34 (31.5)	76 (18.6)	699 (26.5)	809 (25.7)
TNBC	56 (51.9)	165 (40.3)	787 (29.9)	1008 (32.0)
Missing	0	2	6	8
Ki67 index (*n*, %)				
≤ 20%	10 (18.5)	58 (28.9)	438 (33.0)	506 (32.0)
> 20%	44 (81.5)	143 (71.1)	888 (67.0)	1075 (68.0)
Missing	54	210	1315	1579
Grade (*n*, %)				
G1	0 (0.0)	10 (2.4)	88 (3.3)	98 (3.1)
G2	41 (38.0)	186 (45.5)	1305 (49.6)	1532 (48.7)
G3	67 (62.0)	213 (52.1)	1236 (47.0)	1516 (48.2)
Missing	0	2	12	14
pCR				
No	92 (85.2)	376 (91.5)	1954 (74.0)	2422 (76.6)
Yes	16 (14.8)	35 (8.5)	687 (26.0)	738 (23.4)

### Factors associated with the occurrence of CNS metastases as the first site of metastatic disease

In univariate analysis, larger tumor size, node-positive disease, negative hormone receptor status (both estrogen and progesterone negative), poor tumor differentiation (G3 tumor, high Ki67), HER2-positive or triple-negative tumor subtype, and no pCR after neoadjuvant chemotherapy were significantly associated with the occurrence of CNS metastases as the first site of metastatic disease.

Thus, the 5-year cumulative incidence for the occurrence of CNS metastases was 5.92% for patients with a larger initial tumor size (cT3–4) compared to 3.07% for patients with a smaller initial tumor size (cT1–2) (hazard ratio (HR) 2.19, 95% confidence interval (CI) 1.50–3.22, *p* < 0.0001). Patients with a node-positive disease had a 5-year cumulative incidence for the occurrence of CNS metastases of 5.74%. The risk of node-negative patients was 2.07% (HR 3.05, CI 1.98–4.71, *p* < 0.0001). Concerning tumor subtype, a 5-year cumulative incidence for CNS metastases was 4.77% in HER2-positive patients and 6.27% in triple-negative patients. Thus, HER2 positivity was associated with a fourfold and triple negativity with a fivefold higher risk for CNS metastases (HR 4.1, CI 2.06–8.12, *p* < 0.0001 and HR 5.43, CI 2.81–10.47, *p* < 0.0001) compared to luminal A-like tumor subtype.

We additionally evaluated the incidence of CNS metastases among patients with HER2-positive hormone receptor-positive and HER2-positive hormone receptor-negative patients. No significant difference concerning the incidence of CNS metastases could be shown in our cohort of patients between these groups of patients (HR 0.8, 95% CI 0.5–1.22, *p* = 0.268).

The 5-year cumulative risk for development of CNS metastases in patients without achieving a pCR after neoadjuvant chemotherapy was 4.18% vs. 2.49% for patients with a pCR (HR 1.85, CI 1.06–3.24, *p* = 0.0316).

The BRCA status did not show a statistically significant association with the occurrence of CNS metastases as the first site of metastatic disease in our cohort. The data was available for a total of 36 of 108 patients with brain metastases (*n* = 9 BRCA positive, *n* = 27 BRCA negative). Of these patients with known BRCA status, those with a positive BRCA status had a 5-year CNS metastasis incidence rate of 7.84% compared to 4.68% by patients with a negative BRCA status (HR 1.67, 95% CI 0.78–3.57, *p* = 0.189).

In multivariate analysis, risk factors for the development of CNS metastases were larger tumor size (cT3–4; HR 1.63, 95% CI 1.08–2.46, *p* = 0.021), node-positive disease (HR 2.57, 95% CI 1.64–4.04, *p* < 0.001), no pCR after neoadjuvant chemotherapy (HR 2.29, 95% CI 1.32–3.97, *p* = 0.003), and HER2-positive (HR 3.80, 95% CI 1.89–7.64, *p* < 0.001) or triple-negative subtype (HR 6.38, 95% CI 3.28–12.44, *p* < 0.001) (Table 3: multivariate analysis of time to CNS metastases adjusted for non-CNS metastases as competing risk). A multivariate analysis without consideration of pCR status did not show essentially different results.

**Table 3:** Multivariate analysis of time to CNS metastases adjusted for non-CNS metastases as competing risk

Parameter	Hazard ratio	95% hazard ratio confidence limits	Significance level
Analysis without consideration of pCR rate			
Age (continuous)	1.001	0.984–1.018	*p* = 0.9359
Tumor size (cT3–4) (reference category: cT1–2)	1.830	1.223–2.737	*p* = 0.0033
Node-positive disease (reference category: node-negative disease)	2.681	1.710–4.205	*p* < 0.0001
Luminal B-like subtype (reference category: luminal A-like subtype)	1.573	0.611–4.053	*p* = 0.3478
HER2-positive subtype (reference category: luminal A-like subtype)	3.357	1.673–6.736	*p* = 0.0007
Triple-negative subtype (reference category: luminal A-like subtype)	5.569	2.882–10.758	*p* < 0.0001
Analysis with consideration of pCR rate			
Age (continuous)	0.999	0.981–1.016	*p* = 0.8813
Tumor size (cT3–4) (reference category: cT1–2)	1.626	1.075–2.458	*p* = 0.0213
Node-positive disease (reference category: node-negative disease)	2.574	1.641–4.037	*p* < 0.0001
Luminal B-like subtype (reference category: luminal A-like subtype)	1.617	0.627–4.171	*p* = 0.3200
HER2-positive subtype (reference category: luminal A-like subtype)	3.804	1.894–7.639	*p* = 0.0002
Triple-negative subtype (reference category: luminal A-like subtype)	6.384	3.277–12.440	*p* < 0.0001
No pCR after neoadjuvant chemotherapy (reference category: pCR after neoadjuvant chemotherapy)	2.294	1.324–3.973	*p* = 0.0031

We additionally analyzed the incidence of CNS metastases (relating to subtype) among patients without a pCR. A 5-year cumulative CNS metastasis incidence rate in the cohort of patients without a pCR was 1.3% for luminal A-like subtype, 2.53% for luminal B-like, 5% for HER2-positive, and 8.46% for triple-negative patients. The risk of development of the CNS metastases as the first site of metastatic disease was statistically higher in patients with HER2-positive breast cancer as well as for triple-negative patients (HR 3.66, 95% CI 1.78–7.55, *p* = 0.0004 and HR 6.50, 95% CI 3.32–12.71, *p* < 0.0001) compared to patients with luminal A-like breast cancer.

In the cohort of patients with pCR, the median survival was not achieved to the time of evaluation. The 5-year cumulative incidence rates for the patients with pCR after the neoadjuvant chemotherapy were 2.4% for CNS metastases and 5.7% for non-CNS metastases.

## Discussion

CNS metastases have become a major challenge in the management of patients with metastatic breast cancer, as the occurrence of CNS metastases is associated with a particularly bad prognosis. The incidence of CNS metastases in patients that have received neoadjuvant treatment according to current standards has not been reported so far. Only a few reports have examined risk factors for the development of CNS metastases as the first site of metastatic disease in breast cancer patients.

Identification of risk factors for developing CNS metastases would identify a patient group that might benefit from preventive management strategies of CNS metastases, e.g., cranial irradiation or CNS metastasis screening, e.g., by means of magnetic resonance imaging. Both options are not part of the management of patients with breast cancer in clinical routine today. However, the low incidence of CNS metastases as the first metastatic site observed in our cohort and the retrospective design of the study would not justify a screening approach during follow-up. The development of a calculator for the risk assessment could be an aim of the further evaluation of the collected data. On the basis of our analysis, we can recommend to take particular account into neurological complaints of patients with until now not metastasized breast cancer with initially large tumor size, node-positive disease, no-pCR status after systemic therapy, and HER2-positive or triple-negative tumor biology. We suggest carrying out clinical studies investigating the role of screening options (e.g., brain magnetic resonance imaging) for metastatic breast cancer patients who are at high risk for developing CNS metastases.

In our cohort of patients after neoadjuvant chemotherapy, 16% of patients developed metastatic disease after a median follow-up of 5 years. Three percent of patients developed CNS metastases as the first metastatic site. Factors significantly associated with the occurrence of CNS metastases as the first site of metastatic disease were higher tumor stage before therapy (cT3–4), node-positive disease, HER2-positive or triple-negative subtype, and no pCR after neoadjuvant chemotherapy. We could show that tumor subtype remained the highest risk factor for CNS metastases. Our results are predominantly in line with already published analyses of smaller patient cohorts not receiving current treatment regimens.

Gonzales-Angulo et al. [14] determined the incidence of CNS metastases and examined associated disease characteristics in patients with locally advanced breast cancer or inflammatory breast cancer after a neoadjuvant systemic therapy. Five percent of patients (38/768 patients) developed CNS metastases as the first site of metastatic disease. Negative hormone receptor status, nuclear grade 3, positive nodal status, and higher stage of disease were significantly associated with the time to CNS metastasis in this patient cohort.

Other researchers evaluated, in contrary to our design, patient cohorts with either adjuvant systemic treatment or mixed patient cohorts with adjuvant and neoadjuvant approach. Thus, Dawood et al. showed an association of CNS metastases as the first site of metastatic disease and higher breast cancer stage for patients with initially non-metastatic triple-negative breast cancer (*n* = 115) [15]. Patients in this cohort received either adjuvant or neoadjuvant systemic treatment. Atahan et al. observed a correlation between CNS metastases as the first site of metastatic disease and the initial size of the primary tumor and nodal status in 32 patients. These patients received an adjuvant chemotherapy [16].

A different probability of breast cancer subtypes to metastasize into the brain was also described by others [17–20]. Soni et al. and Kennecke et al. detected an association between HER2-positive and TNBC subtype and the development of CNS metastases. Martin et al. evaluated 968 patients with brain metastases at the time of diagnosis of breast cancer and detected that incidence proportions were highest among patients with hormone receptor-negative HER2-positive and triple-negative subtypes [21]. Sihto et al. [19] and Smid et al. [20] showed an association between basal-type cancer and CNS metastases. Lin et al. detected that triple-negative tumors were associated with a greater risk of brain metastases relative to hormone receptor-positive/HER2-negative tumors [22]. Vaz-Luis et al. evaluated 3394 patients with HER2-positive breast cancer and could show that hormone receptor-negative HER2-positive patients were more likely to have recurrence in the brain compared to hormone receptor-positive HER2-positive patients [23].

A noteworthy finding in our study relates to pCR rates in the analyzed subgroups. The pCR rate in patients with CNS metastases as the first site of metastatic disease was higher (15%) compared to patients with other distant metastases as the first site of metastatic disease (9%). Thus, a good response rate to a systemic treatment seems not to have a deciding role in the prevention of CNS metastases. Possibly, more aggressive disseminated tumor cells, which also did not respond to a systemic treatment, gain a propensity to overcome the blood-brain barrier and survive in the central nervous system. Most systemic treatment approaches are assumed not to cross the intact blood-brain barrier; this fact might explain why tumor cells that already reached the CNS cannot be affected. Probably, the initially more aggressive tumor is also a significant risk factor in the development of CNS metastases. In our cohort, 16 patients developed CNS metastases as the first site of metastatic disease despite achieving a pCR. All of them had either HER2-positive (*n* = 9) or TNBC (*n* = 7) primary tumor.

The strength of our analysis is a large cohort of patients who received up-to-date treatment with anthracyclines and taxanes as well as up-to-date HER2-directed therapy. However, given the relatively low incidence of CNS metastases, we cannot identify differences between the incidence of CNS metastases in different treatment groups of the trials, e.g., between lapatinib- and trastuzumab-treated patients with HER2-positive breast cancer or in the cohort of triple-negative patients with carboplatin therapy compared to patients without carboplatin therapy. A small number of patients with an available BRCA status in our cohort also do not allow a confident statistical statement concerning the correlation with the development of CNS metastases and may be regarded solely as trend. A further limitation of our analysis is that the incidence of CNS metastases after the occurrence of metastatic disease was not documented, as in many other trials. This point must be taken into consideration by the interpretation of Fig. 1. The flattening of the curve after 24 months does not prove that CNS metastases do not appear after this period of time as the curve does not include patients that developed CNS metastases after diagnosis of other distant metastases.

## Conclusions

In conclusion, our analysis showed that especially patients with HER2-positive and triple-negative tumors are at risk of developing CNS metastases despite active systemic treatment, especially when no pCR was reached. However, also patients with pCR are at risk of developing CNS metastases. A better understanding of the underlying mechanisms is required in order to develop potential preventive strategies and should help to identify molecular mechanisms of resistance to modern therapies and clonal selection of tumor cells with special brain tropism. Further studies should aim to identify a group of patients that might have a benefit of screening for brain metastases. Further research on biomarkers for CNS metastasis development should be performed. A translational research project in our cohort is ongoing.

## Abbreviations

CI: Confidence interval
CNS: Central nervous system
HER2: Human epidermal growth factor receptor 2
HR: Hazard ratio
pCR: Pathologic complete response
TNBC: Triple-negative breast cancer
